# The dilemma of controlling cultural eutrophication of lakes

**DOI:** 10.1098/rspb.2012.1032

**Published:** 2012-08-22

**Authors:** David W. Schindler

**Affiliations:** Department of Biological Sciences, University of Alberta, Edmonton, Alberta, CanadaT6G 2E9

**Keywords:** lake fertilization, oligotrophication, phosphorus

## Abstract

The management of eutrophication has been impeded by reliance on short-term experimental additions of nutrients to bottles and mesocosms. These measures of proximate nutrient limitation fail to account for the gradual changes in biogeochemical nutrient cycles and nutrient fluxes from sediments, and succession of communities that are important components of whole-ecosystem responses. Erroneous assumptions about ecosystem processes and lack of accounting for hysteresis during lake recovery have further confused management of eutrophication. I conclude that long-term, whole-ecosystem experiments and case histories of lake recovery provide the only reliable evidence for policies to reduce eutrophication. The only method that has had proven success in reducing the eutrophication of lakes is reducing input of phosphorus. There are no case histories or long-term ecosystem-scale experiments to support recent claims that to reduce eutrophication of lakes, nitrogen must be controlled instead of or in addition to phosphorus. Before expensive policies to reduce nitrogen input are implemented, they require ecosystem-scale verification. The recent claim that the ‘phosphorus paradigm’ for recovering lakes from eutrophication has been ‘eroded’ has no basis. Instead, the case for phosphorus control has been strengthened by numerous case histories and large-scale experiments spanning several decades.

## 1. Introduction

Cultural eutrophication is the Earth's most widespread water quality problem. It causes harmful algal blooms, fish kills and many related problems both in fresh waters and in coastal seas that are adjacent to areas with large human populations [1]. Most scientists agree that it is largely caused by increasing inputs of phosphorus and nitrogen, which are abundant in human sewage, in the excrement of livestock and in synthetic fertilizers applied to agricultural land.

There is less agreement on how to reverse cultural eutrophication (*oligotrophication*). Whole-lake experiments showed that addition of phosphorus was essential to creating algal blooms [2,3]. As a result, phosphorus inputs to many culturally eutrophied lakes were reduced, and there are now many case histories showing successful reversals of lake eutrophication [1].

In contrast, most estuarine scientists have argued that it is necessary to reduce nitrogen instead or as well as phosphorus, based on bottle bioassays, mesocosms, nutrient ratios and other short-term indicators of nitrogen limitation (reviewed by Howarth & Marino [4]). There are also case histories where phosphorus control has not reduced eutrophication in estuaries. The European Union has required inputs of both elements to be reduced to the Baltic Sea and other coastal waters via its Water Framework Directive (http://ec.europa.eu/environment/water/water-framework/index_en.html). Similar recommendations have been made for coastal waters in the USA.

Recently, several authors have applied similar reasoning to argue that it is also necessary to reduce nitrogen loading to reverse eutrophication of lakes [5–9]. One study, by Lewis & Wurtsbaugh [5], concluded that the phosphorus ‘paradigm’ has been ‘eroded’. Meanwhile, the success of ‘P only’ control in lakes is widely documented and recognized (table 1).

**Table 1.** Lakes that recovered from eutrophication following the control of phosphorus inputs (partial list).

Lake Erie	Lake Ontario
Lake Geneva, Switzerland	Lake Lucerne, Switzerland
Lake Zurich, Switzerland	Lake Constance, Switzerland
Lago Maggiore, Italy	Lake Norrviken, Sweden
Lake Malaren, Sweden	Lake Hjalmaren, Sweden
Lake Vattern, Sweden	Lake Vanern, Sweden
Gravenhurst Bay, Ontario	Little Otter Lake, Ontario
Meretta Lake, Canada Lake Washington,	Kootenay Lake, British Columbia
Washington	Moses Lake, Washington
ELA lakes—226 NE, 303, 304, 261	Lake Balaton, Hungary
Stockholm Archipelago (brackish water)	

The controversy over whether reversing cultural eutrophication requires reducing inputs of nitrogen, phosphorus or both has important financial implications in both lakes and coastal waters. Controlling sources of both elements to meet the European Union's Water Framework Directive in the Baltic Sea would cost 3.1 billion euros. Controlling phosphorus alone would cost only 0.21–0.43 billion euros [10]. Similarly, removing both elements from sewage in the city of Winnipeg, Manitoba, Canada is estimated to cost from four- to eightfold more than removing phosphorus alone [11]. Some believe that costly controls of nitrogen in land run-off may impair crop production, compromising human food supplies [12]. Poorer jurisdictions may not be able to afford to reduce sources of both elements. It is therefore important to deduce when eutrophication can be reduced by controlling phosphorus alone, and when more expensive control of nitrogen may be required.

In the following review, I find that three types of errors have affected many of the scientific recommendations: (i) It is assumed that short experiments where nutrients are added to small bottles or mesocosms can be extrapolated to whole ecosystems over long time periods. (ii) Conclusions about reversing eutrophication are made by *adding* nutrients to water rather than *decreasing* nutrients. (iii) There are flawed assumptions and logic about ecosystem-scale nutrient cycling. I also review the success of several whole-lake experiments and case histories of adding or removing phosphorus and/or nitrogen individually and in combination.

## 2. Extrapolating from measures of proximate nutrient limitation to ecosystem scales

It is usually assumed that if a nutrient stimulates algal abundance or production in short, small-scale experiments it will also control the abundance and production of phytoplankton in lakes over long periods. Comparing short experiments with whole-lake nutrient additions, Schindler [13] and others [14,15] concluded that such extrapolations were often misleading, but reliance on bottle bioassays and mesocosms has continued. Ecosystem responses include many slow, long-term changes to nutrient fluxes, and species successions that cannot be assessed by short, small-scale experiments [16–18].

Recently, Vitousek *et al*. [19] proposed that short-term nutrient limitations be designated as *proximate*, whereas nutrients that provide long-term controls on ecosystem productivity and plant biomass be termed *ultimate* limiting nutrients, concluding that measures of proximate nutrient limitation do not necessarily provide useful information about ultimate nutrient limitation. Reducing eutrophication is a long-term problem that always requires control of the ultimate limiting nutrient.

### (a) Bottle bioassays

Nutrients are added to algal cultures or bottles of unfiltered lake water, and the resulting changes in algal biomass or photosynthesis are measured after a few hours to a few days. Short-term bottle- and mesocosm-scale experiments showing carbon limitation were used by others as ‘proof’ that controlling phosphorus would not be effective in controlling lake eutrophication, but comparison of bottle bioassays with long-term whole-lake experiments showed that the bottles did not account for the invasion of CO_2_ from the atmosphere, which in lakes supplied enough carbon to allow algal biomass to increase in proportion to phosphorus. Over several years, atmospheric invasion and recycling corrected the lake's carbon deficiency [1,3,16].

In later years, short bioassays in Lake 227 often showed nitrogen limitation in early summer, followed by the appearance of nitrogen-fixing heterocystous Cyanobacteria, after which the algal community returned to phosphorus limitation [20]. Short-term nutrient bioassays in such cases are misleading, because even nitrogen-fixing Cyanobacteria will preferentially take up ionic nitrogen if it is available, although they can eventually achieve the same biomass without it. In an individual year, fixation of atmospheric nitrogen supplied only a small part of the nitrogen requirements of algae in Lake 227, but successive years of fixation and recycling of sedimented nitrogen slowly increased the lake's biogeochemical pools of nitrogen. Together, fixation and recycling met long-term algal demands for growth at ecosystem scales [17,18].

Recently, meta-analyses of small-scale nutrient experiments from many lakes revealed that adding nitrogen plus phosphorus almost always results in greater algal biomass than adding either nutrient alone [21]. It was concluded that nitrogen deposition is causing previously nitrogen-limited lakes to become phosphorus-limited [22]. This seems highly unlikely, because lakes in similar geological settings in northern North America, where nitrogen deposition is very low, are phosphorus-limited. The average duration of a single study included in the meta-analysis was only 7 days, and the result is not surprising as proximate responses. Some have argued that this result means that reducing nitrogen loading as well as phosphorus will result in more rapid declines in eutrophication in whole lakes [9,23]. Recently, a 7-day experiment in mesocosms of various sizes was compared with the above-mentioned meta-analyses, concluding erroneously that short-term ecosystem results can therefore be scaled to whole lakes [24], even though no whole-ecosystem measurements were included, and ecosystem processes such as nitrogen fixation and recycling were ignored. Meta-analyses do not improve short experiments as measures of ultimate nutrient limitation.

Proximate nitrogen limitation is more likely to indicate that a eutrophic lake has been over-fertilized with phosphorus than that nitrogen must be controlled. If a group of limnologists armed with standard bottle bioassays and mesocosms were to visit Lake 227 in midsummer, unaware that the lake has been fertilized with phosphorus alone for the past 22 years [17], their results would show extreme nitrogen limitation. Using the logic usually applied, they would recommend that nitrogen loading must be reduced to control eutrophication, clearly absurd for a lake that receives no anthropogenic nitrogen! Similarly, in Moses Lake, Washington, following reduced loading of phosphorus and nitrogen, N limitation was observed in small-scale bioassays for several years, but phytoplankton declined in proportion to phosphorus [25].

In summary, bottles exclude many nutrient fluxes and algal successions that determine the ultimate response of a lake to changes in nutrient supply.

### (b) Mesocosm experiments

Mesocosms have been used, either *in situ* or *in vitro*, for nutrient experiments, assuming that their larger volume and longer duration may better simulate what happens in real ecosystems. We have performed *in situ* mesocosm experiments in parallel with long-term whole-lake experiments at Experimental Lakes Area (ELA), to test their reliability.

Our early blunders with proper scaling of the size and shape of mesocosms to represent limnetic and benthic processes caused them to produce results unlike the responses of whole lakes [13]. In order to get nitrogen-fixing Cyanobacterial blooms in mesocosms similar to those observed in fertilized whole-lakes with low N : P ratios, a thermocline was necessary to prevent nitrogen released from sediments from rapidly returning to the euphotic zone [26,27].

Others have also noted problems with extrapolating from mesocosms to whole lakes. Phytoplankton in a very large (10 000 m^3^) mesocosm in Blelham Tarn slowly declined when compared with the lake, because the mesocosm wall prevented entry of nutrient inputs from the catchment [28]. Higher vertical mixing rates have been observed in mesocosms than in the main lake in which they were installed, the result of wave energy imparted to the mesocosm walls [29].

Mesocosm experiments are difficult to maintain for long periods, and experiments usually last for a few weeks at most. If properly scaled, they can accurately simulate some early features of lake responses, such as early algal succession, and atmospheric and sediment–water exchange, but they still inadequately assess multi-year corrections to biogeochemical processes and nutrient pools, or the effects of changes in populations of piscivorous predators. Mesocosm results must still be regarded as measures of proximate nutrient limitation, and hence of limited use for predicting long-term responses in whole lakes.

### (c) Experimentally adding nutrients to predict the result of removing nutrients

Much of the evidence used to predict the result of *decreasing* nutrients is from experimentally *adding* nutrients to lakes or small containers. Often, recoveries from nutrient loading are confounded by hysteresis [30]. There are also often lags in the response of algal communities [31], perhaps the result of the delayed response of higher trophic levels to reduced primary production. If macrophytes have disappeared owing to shading by dense algal blooms, restoring the original conditions can be difficult [32] (although some lakes also form rapidly changing unstable states [33]). In some cases, hysteresis can delay full recovery of lakes by 10–40 years [25,34,35].

The usual reason identified for hysteresis is the return of phosphorus from sediments enriched by years of high phosphorus input (the so-called internal loading). In polymictic eutrophic lakes with low iron, 90 per cent of the summer input of phosphorus can be internal, and sediments can remain the dominant phosphorus source for several years after external loading is reduced, though it slowly declines in importance [25,34,36]. High concentrations of inorganic phosphorus after loading is decreased are a sign that light, not nutrients, may be limiting the lake's phytoplankton. In many cases, where decreasing external phosphorus loading appears to be ineffective, monitoring after reduction in loading may not have been long enough to detect recovery [25]. There can be other complications. Köhler *et al*. [37] found that reducing N loading caused lower nitrate levels, leading to an increase in internal loading of phosphorus in a shallow German lake, thereby delaying recovery. Presumably, this was because nitrate functioned as an electron acceptor, delaying the release of phosphorus at the mud–water interface. As in Lake 227 [17], they found that reducing N loading did not reduce the midsummer abundance of N-fixing Cyanobacteria.

Saturation of catchment soils with phosphorus may also slow a lake's recovery after point sources are controlled. This often occurs where manure is applied in a quantity sufficient to satisfy the demands of growing crops for nitrogen. Because manure has a much lower N : P ratio than harvested plants, excess phosphorus is left in the soil. High phosphorus level in run-off may take years or even decades to subside [38,39]. Intensive application of nitrogen in catchments already saturated with phosphorus has been reported to cause nitrogen-fixers to be replaced by other Cyanobacteria such as *Planktothrix* and *Microcystis* spp. [40]. The presence of greater than 100 µg l^−1^ of phosphate, nitrate and chlorophyll *a* suggests that such systems are nutrient-saturated and phytoplankton are probably limited by light. The earlier-mentioned genera thrive under low light conditions [41,42]. Controlling nitrogen in such lakes would probably lead to a return of nitrogen fixers, whereas controlling phosphorus would lead to a loss of excess nitrate through denitrification, followed by replacement of Cyanobacteria by chlorophytes, chrysophytes and diatoms, and declines in algal abundance. Phytoplankton in degraded lakes are unlikely to decline until total phosphorus is less than 50 µg l^−1^, and phosphate is nearly undetectable.

### (d) Errors in the interpretation of ecosystem processes and indicators

#### (i) Error 1: assuming that annual N fixation must supply the entire annual nitrogen demand of plankton

Many authors have assumed that to be effective, annual nitrogen fixation must account for the entire annual nitrogen requirement for algal production [4,5,8,23]. In lakes, this is not the case [17,18]. Fixed atmospheric nitrogen is subject to the same efficiencies of sedimentation and in-lake recycling as other nitrogen sources. Lake 227 has a moderate water renewal time (about 4 years); so a high proportion of fixed atmospheric nitrogen is sedimented with algal remains and recycled. Contrary to the unsubstantiated claims of Scott & McCarthy [23], denitrification losses are low. This was the case even when the lake was fertilized with high N : P ratios in the early 1970s [43]. Annual nitrogen requirements of phytoplankton are met by the combination of fixation, return from sediments, atmospheric deposition and run-off. As a result, nitrogen concentration increases over several years [16,17]. Accretion and recycling of fixed nitrogen can eventually exceed total N loading from external sources.

As we found in Lake 227, in tropical Lake Victoria, nitrogen fixation supplied only 20 per cent of the daily N demand by phytoplankton, but fixed nitrogen accounted for 70 per cent of annual loading of total nitrogen [44].

#### (ii) Error 2: assuming that ratios of total, suspended or dissolved nutrients are reliable indicators of nutrient limitation

Phytoplankton in both fresh waters and marine systems always have N : P ratios rather close to Redfield ratios, regardless of which nutrient might be limiting [15]. Because particulate matter is usually 70 per cent or more of the total nutrient pool, the ratio of total N : P is a very poor indicator of nutrient limitation. In our whole-lake experiments, despite widely varying ratios in inputs of phosphorus, nitrogen and carbon, the ratios of concentrations of the three elements varied very little [3]. Some have used the relative strength of the chlorophyll *a* : total phosphorus correlation and the chlorophyll *a* : total nitrogen correlation as a measure of which nutrient is more likely to be limiting. Typically, both nutrients are strongly correlated to each other, as Redfield ratios suggest they should be, and to chlorophyll *a.* In short, all three are largely measures of the same thing (i.e. collinear). Collinear predictors affect the interpretation of many environmental studies [45]. Slightly better correlations of one nutrient or the other with chlorophyll *a* are, in my experience, more likely to reflect relative differences in analytical errors than in nutrient limitations.

#### (iii) Error 3: misinterpreting or misrepresenting older Experimental Lakes Area experiments

Lewis & Wurtsbaugh [5] constructed a figure from early data for fertilized ELA lakes, first published over 30 years ago [46]. They concluded correctly that lakes in the table that received both nitrogen and phosphorus produced higher chlorophyll *a* concentrations than those fertilized with phosphorus alone, which is not surprising given the long time required for nitrogen to reach a steady state when the element is in short supply, as discussed earlier. They incorrectly concluded that the lake fertilized with nitrogen alone (Lake 226 SW) responded by increasing algal chlorophyll *a* concentrations, ignoring the evidence for leakage of phosphorus from Lake 226 NE, which was fertilized with phosphorus and nitrogen. The basins were separated only by a plastic curtain, which leaked on several occasions. A forest fire in the catchment supplied additional phosphorus [47,48]. Leakage of nutrients around a similar curtain used to isolate basins of a lake was documented using stable nitrogen isotopes [49]. In general, curtains are not sufficient to isolate basins of a lake if multi-year comparisons are required.

The above-mentioned figure has other flaws. Only 4 years of data (1973–1976) were included for each of six fertilized lakes. For two of the lakes, the 4 years included 1 or 2 years of either pre- or post-fertilization data. For another lake, data included 2 years of high N : P fertilization followed by 2 years with low N : P. Even for the three lakes fertilized in all 4 years, they were the first few years of fertilization, when less than full responses would be expected from lakes where nitrogen was in short supply [16,17]. The lakes also received different loading rates of phosphorus, so, even if all were at steady state, different chlorophyll *a* concentrations would be expected.

The flawed figure was cited [9,23] as evidence that nitrogen as well as phosphorus must be controlled to reverse eutrophication. More data are available for fertilized ELA lakes than was the case in 1979. Below, I analyse the full period of fertilization and recovery for lakes in the study of Lewis *et al.* [9], and results from a lake fertilized with only nitrogen.

## 3. Manipulation of nutrient inputs to whole lakes

Long-term, whole-ecosystem nutrient manipulations are the only reliable way to evaluate the long-term effectiveness of nutrient addition and removal. Experiments at sizes smaller than whole lakes appeared to lack some of the key processes that affect ecosystem-scale responses. On the other hand, while many features of lakes change with size, it is relatively simple to scale up physical, chemical and biological processes to predict the responses of large lakes from results in smaller ones [50].

### (a) Evidence from experimental additions and reductions of nutrients to whole-lakes

Whole-lake experiments have been conducted at several locations in North America and Europe.

#### (i) Experimental fertilization of lakes with phosphorus alone

**Figure 1.**
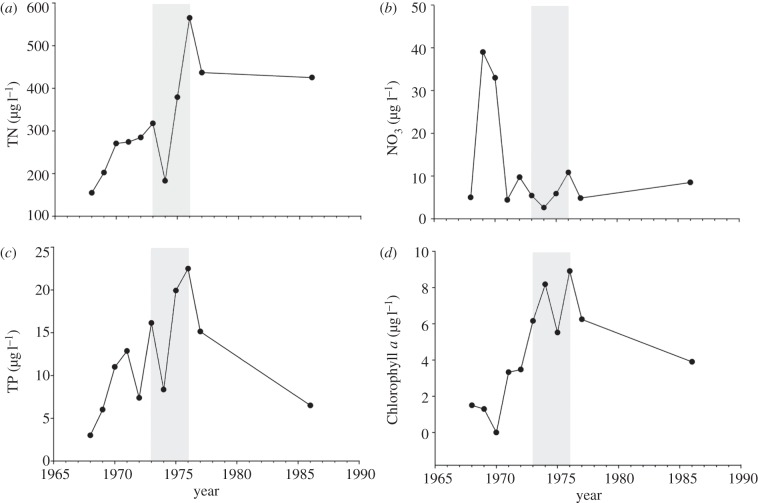
(*a*) Total nitrogen (TN), (*b*) nitrate, (*c*) total phosphorus (TP) and (*d*) chlorophyll *a* in Lake 261 of ELA, before, during and after experimental fertilization with phosphorus, designated by the shaded area. Values are means for the epilimnion in the ice-free season. Note the successive increases in TN and chlorophyll *a*, despite the fact that only phosphorus was added to the lake.

Lewis & Wurtsbaugh [5] concluded that because adding P alone to Lake 261 at ELA produced lower chlorophyll *a* concentrations than ELA lakes receiving both N and P, nitrogen was necessary for eutrophication. This ignores two factors: first, Lake 261 was fertilized with phosphorus at much lower rates than other fertilized lakes. As a result, a lower response would be expected even after full steady state had been reached. Second, because nitrogen inputs from natural sources such as N fixation and recycling from sediments increase very slowly, the first 4 years of fertilization are not representative of a full steady-state response in the lake [16]. In Lake 261, nitrogen fixation in summer was high in the epiphyton on stalks of *Nuphar variegatum*. Phytoplankton and nitrogen increased gradually, at least doubling over the 4 years of fertilization (figure 1), probably as the result of recycling of nitrogen fixed by epiphytic Cyanobacteria. If nitrogen increases were as slow as in Lake 227 [16], steady-state concentrations of nitrogen and phytoplankton would not have been reached by the final year of fertilization.

Phosphorus was added to Far Lake, an oligotrophic lake well north of the tree line on the west coast of Hudson's Bay [51]. Increased fixation of nitrogen occurred in the epilithon. As in Lake 261, concentrations of total nitrogen increased. *Anabaena* appeared in the plankton of the lake, whereas it was absent in pristine lakes of the area, except for coastal lakes in the area that were periodically inundated by the sea [52]. Again, fertilization was only for 3 years, and it is unlikely that the phytoplankton had responded fully.

Hymenjaure in the Swedish subarctic was fertilized with phosphorus [53,54]. In the first 2 years of fertilization, increases were noted in epilithic nitrogen fixation, total nitrogen and the appearance of nitrogen-fixing species. Again, the period of fertilization with P (3 years) was too short to expect a full response. Fertilization of lakes in Labrador for 3 years caused plankton chlorophyll *a* to increase in only one lake [55]. Nitrogen fixation and nitrogen concentrations were not measured, so biogeochemical changes cannot be compared with the above-mentioned lakes.

In summary, where biogeochemical processes are measured, whole-lake experiments in the global north respond to phosphorus alone. Nitrogen fixation allows slow increases in nitrogen concentration and phytoplankton. No experiment was carried out long enough to explore the full response to phosphorus alone. Extremely high rates of nitrogen fixation on rocky substrates were found in Lake Malawi [56], even while pelagic rates were low [57], suggesting that the initial response to phosphorus enrichment may be in littoral–benthic zones, and that lakes in warmer regions may respond more rapidly.

Lake 227 was fertilized with nitrogen and phosphorus for 21 years, followed by 22 years of fertilization with phosphorus alone. Nitrogen fixation increased after nitrogen fertilization ceased, and there was no decline in phytoplankton [17,18]. This supports the above-mentioned results, suggesting that, with time, a standing crop would develop in response to phosphorus alone that would equal the response to both phosphorus and nitrogen.

#### (ii) Experimental fertilization of lakes with nitrogen alone

**Figure 2.**
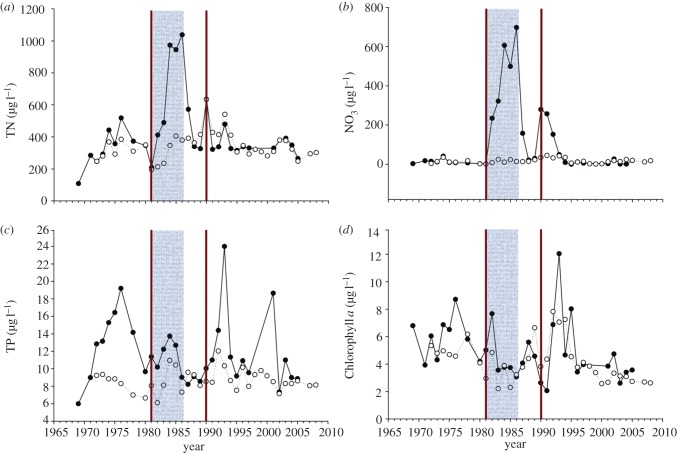
The same parameters as in figure 1, for Lake 302 N (solid line, black circles), to which nitric acid was added, and Lake 302 S (dotted line, white circles), which received sulphuric acid. Neither basin received phosphorus. The period of addition is designated by shading (302 N) and two vertical lines (302 S). Note the lack of discernible response of chlorophyll *a* to nitrogen input.

Nitric acid was added to Lake 302 N at ELA for 6 years (1981–1986). The adjacent Lake 302 S received an equivalent amount of sulphuric acid per unit volume to compare acidification by inputs of sulphuric and nitric acids, but the pH decrease was not enough to affect algal biomass because of internal alkalinity generation. There was no evidence of any fertilizing effect of nitrogen (figure 2). Concentrations of chlorophyll *a* were similar in the two basins. Occasional blooms of chrysophyceans occurred in the metalimnions of both basins before, during and after the experiment [58].

Nitrate accumulated in Lake 302 N to concentrations of several hundred µg l^−1^ during nitrate addition (figure 2), despite 40-fold increases in denitrification [59]. By contrast, nitrate and denitrification remained near undetectable levels in Lake 302 S. After addition of nitrate ceased, nitrate concentrations returned to pre-fertilization values quickly, although a small 3-year increase in nitrate followed a few years after the experiment was terminated (figure 2).

In northern Sweden, Lake Magnusjaure was fertilized with nitrogen for 3 years. Initially, phytoplankton increased several-fold, then remained stable [53]. No explanation is given for the response, and it may reflect natural variation. However, as an anonymous reviewer pointed out, ammonium nitrate from agricultural suppliers is sometimes contaminated with phosphorus, which is a possible explanation. When phosphorus was deliberately added as well as nitrogen in the final year of the experiment, much higher phytoplankton biomass was reached.

#### (iii) Experimental fertilization of whole lakes with both phosphorus and nitrogen

Rapid increases in phytoplankton occur when both phosphorus and nitrogen are added, unless the system is already nutrient-saturated or light-limited, but slower increases occur when ionic nitrogen is not sufficient to meet algal demands. When N : P is high, taxa already present in plankton increase [52,54,60,61]. Where nitrogen additions have N : P ratios less than Redfield ratios, species that fix atmospheric nitrogen dominate [3,17,44]. While the response of the latter lakes is slower, phytoplankton blooms will eventually develop in proportion to phosphorus concentrations [3,16].

#### (iv) Reducing phosphorus inputs, with and without reducing inputs of nitrogen

**Figure 3.**
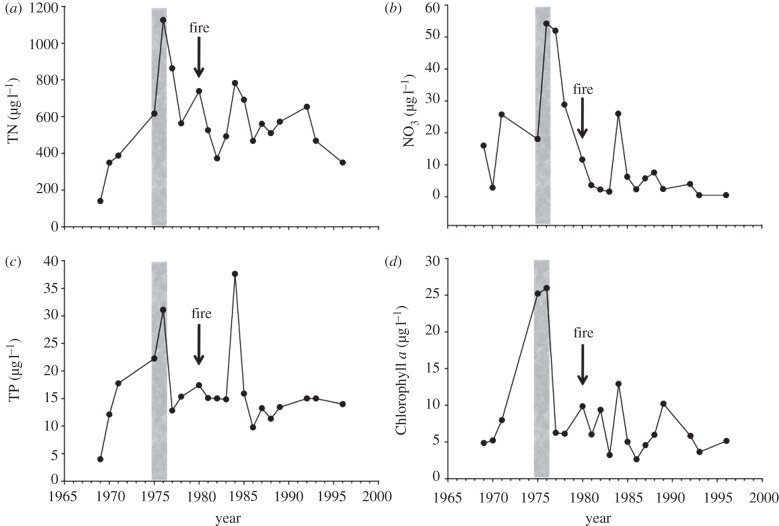
The same parameters as in figure 1, for Lake 303, fertilized with nitrogen and phosphorus at a ratio of 15 : 1 (weight), as designated by the shaded area. Note the rapid increase in chlorophyll *a*, followed by a decrease in proportion to the decline in phosphorus, while excess nitrogen accumulates as nitrate. The arrow designates a forest fire in the catchment, which burned most of the vegetation and organic soil.

Lake 303 was fertilized with phosphorus and nitrogen at a 1 : 15 ratio by weight for 2 years, causing a rapid increase in chlorophyll *a* (figure 3). After fertilization ceased, phosphorus and chlorophyll *a* returned rapidly to near background. Excess nitrogen accumulated as nitrate, but was removed by denitrification a year later (figure 3). Dissolved organic nitrogen remained high for several years [62].

**Figure 4.**
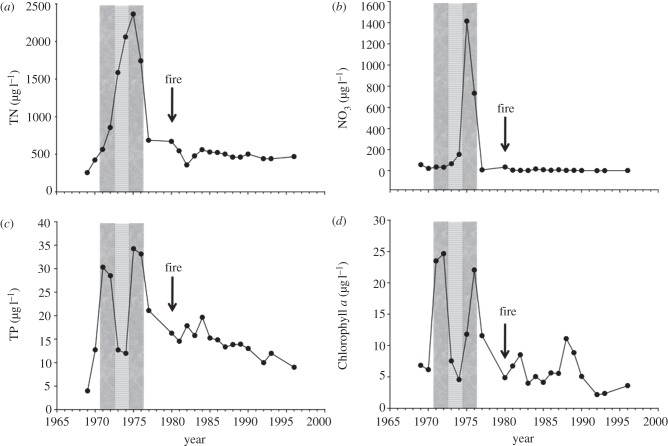
The same parameters as in figure 1, for Lake 304. The lake was fertilized with phosphorus, ammonium and sucrose in 1971–1972, ammonium and sucrose but no phosphorus in 1973–1974, then phosphorus and nitrate in 1975–1976. Note that the chlorophyll *a* increases rapidly when phosphorus is added, and decreases rapidly when it is not, whether or not nitrogen is added. The arrow designates a forest fire that burned most of the catchment.

Lake 304 was fertilized with phosphorus, ammonium chloride and sucrose in 1971–1972, causing a phytoplankton bloom (figure 4). In 1973–1974, nitrogen and carbon were added, but not phosphorus. Phosphorus, phytoplankton abundance and species composition returned rapidly to near pre-fertilization values, and inorganic nitrogen accumulated in the lake. In 1975–1976, phosphorus was again added, with sodium nitrate. Chlorophyll *a* concentrations increased once again, in proportion to phosphorus. The lake recovered rapidly after fertilization ceased (figure 4).

In summary, phytoplankton in all of the fertilized lakes at ELA recovered rapidly from eutrophication, in proportion to phosphorus, regardless whether nitrogen inputs were terminated. Nitrogen accumulated as nitrate after phosphorus input was reduced, and was lost by denitrification [59,62].

Lake N-2 in northern Alaska was split with a sea curtain, and half the lake was fertilized for 6 years with both phosphorus and nitrogen. After fertilization ceased, the phytoplankton declined rapidly in proportion to phosphorus, but nitrogen flux from sediments remained high and bottom water remained anoxic [63].

### (b) Evidence from case histories where nutrient inputs to lakes have been reduced to manage cultural eutrophication

#### (i) Delayed response to phosphorus control: hysteresis leads to confusion over which nutrient to control

As mentioned earlier, early management efforts to decrease eutrophication in lakes focused heavily on controlling phosphorus, and it was usually successful (table 1). In some cases, recovery took many years, as the result of internal loading of phosphorus, and possibly slow reductions in non-point sources such as phosphorus-saturated soils, as discussed later. In a review of 35 case studies, decreasing external loading of phosphorus usually reduced in-lake concentrations of total phosphorus and phytoplankton chlorophyll *a*, and increased water clarity [35]. In most lakes, a new steady state was reached in 10–15 years after inputs were decreased. The rate of decline was not affected by water renewal time, and internal phosphorus loading appeared to be the main reason for the delay. In some of the lakes, nitrogen loading was reduced as well, but it is difficult to discern any difference in rate of response from lakes with phosphorus control alone.

Many of Europe's largest lakes have been recovered from eutrophication by rigorously reducing external loading of phosphorus. I will mention a few specific cases. The Lake of Constance (Bodensee) was one of Europe's most ultraoligotrophic lakes in the early twentieth century. Rapid industrialization and urbanization in the lake's catchment in mid-century increased the phosphorus load considerably, causing eutrophication. Stringent phosphorus controls were introduced in the early 1980s, and by 2007, phosphorus had declined almost to background values, with chlorophyll *a* following in proportion, despite the tendency of warming climate to make the lake more productive [64].

Lago Maggiore, Italy, was the site of an aggressive campaign to reduce phosphorus inputs after 1977, when the lake had changed from its original oligotrophic state to mesotrophic. The lake had recovered to nearly pristine conditions by the 1990s [65]. Phosphorus control has been less successful in other sub-alpine Italian lakes, although some are showing preliminary signs of recovery [65].

From 1970 to 2000, phosphorus concentrations declined in 11 European alpine lakes where input of phosphorus was decreased [66]. Fish production also declined, and there was a slow shift from cyprinid fishes, which dominated at higher eutrophication, to the coregonid species present before eutrophication. While phytoplankton data were not presented, changes in fish production were attributed to reduced phosphorus.

In Sweden, lakes Norvikken and Mälaren recovered after point source phosphorus was decreased [34,67].

In apparent contrast, reducing phosphorus loading caused no detectable reduction in phytoplankton after 12 years in shallow Little Mere, UK. Phosphorus concentrations remained high. Despite low N : P ratios, nitrogen-fixing Cyanobacteria were not conspicuous. Recycling of nutrients from sediments appears to be high, and there was no declining trend [36].

In deeper Rostherne Mere, phosphorus declined at least threefold after sources were reduced, but there was no corresponding decline in phytoplankton. However, greater than 150 µg l^−1^ of phosphorus remained 12 years after loading was reduced, most of it as soluble reactive phosphorus (SRP). Such high concentrations of phosphate probably indicate that the system was limited by light. Regression showed slow linear declines in total P and SRP [36]. If these trends held beyond the period of record, phytoplankton should now be declining in proportion to phosphorus. Moss (B. Moss 2011, personal communication) has been unable to continue monitoring the system after his retirement. He speculates that owing to continued agricultural use of fertilizer in the Mere's catchment, diffuse nutrient sources probably caused phosphorus concentrations to level off at values where water quality will still be unacceptable. Rostherne Mere is an important case history to assess whether catchment-wide nutrient controls are necessary to recover lakes from eutrophication. Longer monitoring would tell whether phosphorus use by agriculture must be regulated, or whether the current phosphorus controls will eventually be sufficient.

In a Wisconsin watershed subjected to decades of heavy fertilization with phosphorus, Carpenter [38] calculated that several decades would be required for new steady states to be reached.

Two other North American recovery case histories, Lake Washington and Moses Lake, also showed strong hysteresis. In both cases, chlorophyll *a* declined over several years, in proportion to the decline in phosphorus concentration. As found in ELA lakes, excess nitrate accumulated in the lakes, eventually to be lost by denitrification [25,68].

The recoveries of Lake Arresø, Denmark and Lake Paranoa, Brazil were studied after decreased loading of phosphorus and nitrogen [69]. Reductions in phytoplankton generally paralleled declining concentrations of phosphorus, but it is unclear whether reductions in nitrogen had an additional effect. In both cases, algal blooms remained too high to allow the recovery of macrophyte beds that were shaded out during the hypereutrophic conditions. It has been suggested by others that restoring macrophyte beds in recovering lakes might require excluding fish and birds from the recovering areas [37].

In brief, long periods of hysteresis can occur as the result of internal loading, high phosphorus in catchment soils and slow recovery of fisheries. These must not be confused with the need for controlling nitrogen or other measures.

Simultaneously decreasing inputs of both phosphorus and nitrogen before it is known whether controlling phosphorus alone will be unsuccessful can lead to an expensive ambiguity, as it will not be clear whether the response of the lake has been caused by reductions in both nutrients, or if phosphorus alone would have been adequate.

### (c) Controlling symptoms of eutrophication by adding nitrogen

Nitrogen has been added to lakes to prevent the appearance of nitrogen-fixing Cyanobacteria. In hypereutrophic prairie pothole lakes, nitrogen-fixing Cyanobacteria are very common in summer, due to high internal phosphorus loading and polymixis. Often, the blooms collapse in late summer, and their rapid decay causes total anoxia or ‘summerkill’ in the lakes, preventing aquaculture of trout [70]. Nitrogen fixers (chiefly *Aphanizomenon flos-aquae*) were suppressed by adding ammonium nitrate to a small lake, and to mesocosms in other prairie lakes. To successfully suppress *Aphanizomenon*, the nitrogen had to be added early in the summer, before the blooms started to appear. The effects of the addition only lasted one year, suggesting that repeated additions would be necessary [71,72]. Adding nitrate has been used to prevent nitrogen fixers from dominating the communities of freshwater ponds. A part of the effect is due to nitrate's role as a terminal electron receptor, preventing phosphorus release from sediments [73].

Reducing phosphorus while keeping nitrogen loading high has been successful in suppressing nitrogen fixers in Lake Onondaga, New York [74,75]. The form of nitrogen had to be carefully controlled to prevent anoxic events at fall overturn, when nitrification was high. Occasional toxic concentrations of ammonium and nitrite were also encountered. The strategy did not prevent the appearance of Cyanobacteria species that were not N fixers.

Nitrate was added to Indian Lake in an unsuccessful attempt to shift the algal bloom away from non-nitrogen-fixing *Microcystis* [76].

In brief, several lines of evidence suggest that decreasing inputs of nitrogen will not hasten recovery from eutrophication, and may even hinder it by favouring nitrogen fixers or stimulating internal loading of phosphorus.

### (d) Hydrology, eutrophication and oligotrophication

Many models of climate change predict either increases or decreases in precipitation, depending on regional patterns of atmospheric circulation. The resulting changes in hydrology can affect eutrophication from point sources of nutrients [77,78]. In contrast, where non-point sources predominate, increasing inflow can increase nutrient input to the system, via run-off from nutrient sources in a lake's catchment. Such inputs can be highly episodic, causing high seasonal and interannual variation in loading [11]. In a dry year, much of the nutrient remains in soil, because little run-off occurs during the summer months. However, run-off can be much higher in the occasional wet years, when spring floods and large rainstorms mobilize nutrients into streams and groundwaters. For example, slight increases in precipitation have greatly increased the frequency of flooding of the Red River, the main nutrient source to Lake Winnipeg, Canada, resulting in a near doubling of eutrophication in less than two decades [79].

## 4. Some changes in thinking are necessary

Changes are necessary in how nutrient limitation is assessed and interpreted if we are to successfully control eutrophication of lakes. We must rely more on long-term, ecosystem-scale experiments and real case histories, and less on small-scale experimentation, which, although more precise, ignores ecosystem processes that are vital to the response of whole lakes to phosphorus loading. We must cease assuming that the effect of nutrient *additions* to lakes necessarily enables us to predict what will happen when nutrients are *decreased*. Case histories of lakes after nutrient controls are in place will allow us to learn more about the mechanisms and duration of hysteresis.

Evidence at ecosystem scales suggests that decreasing nitrogen sources will have either adverse consequences (increased blooms of nitrogen-fixing Cyanobacteria, possibly enhanced phosphorus return from sediments) or no effect on the oligotrophication of lakes.

### (a) Do the above-mentioned observations apply to coastal waters?

After decades of debate, there is still controversy about how to reduce eutrophication of estuaries. In contrast to my conclusions for lakes, Howarth & Marino [4] concluded that bioassays and mesocosms accurately reflect responses at ecosystem levels in estuaries. But the Marine Ecosystems Research Laboratory mesocosms were the largest scale reviewed, and the longest experiments were only for several weeks. In lakes, these would be far too short to assess the long-term ecosystem-scale processes that we have found to be important in keeping the response of algae proportional to phosphorus.

Despite recent claims that reducing nitrogen is essential to curbing estuarine eutrophication, there is no documented case history of where this has been successful. There is one documented case of recovery of a low-salinity estuary, the Stockholm Archipelago, where eutrophication decreased considerably as a result of decreased phosphorus loading. As observed in lakes, nitrogen-fixing Cyanobacteria and nitrogen fixation declined as the N : P ratio of the system increased from 7 : 1 to more than 26 : 1 over several years. As in lakes, the decline in chlorophyll *a* was in proportion to the decline in phosphorus, despite the summer phytoplankton being nitrogen-limited [80,81].

In Himmerfjärden, a brackish (6 psu) coastal inlet off the west coast of Sweden, reducing nitrogen inputs relative to phosphorus allowed *Aphanizomenon* to become dominant, even though concentrations of both nutrients and chlorophyll *a* were very low [81]. This is again consistent with observations in lakes.

These observations suggest that estuaries with low salinities, long water residence times, abundant nitrogen-fixing Cyanobacteria and high denitrification will respond to phosphorus control much as lakes do.

Almost all of the evidence used to argue for nitrogen control in estuaries is subject to the same criticisms as I have applied to lakes mentioned earlier. Given the enormous expense involved in controlling nutrient inputs to large coastal seas such as the Baltic, it seems prudent to perform long-term ecosystem-scale experiments of the sort that provided essential information in lakes before requiring widespread control of nitrogen in sewage and run-off. In contrast, there are good reasons to control nitrogen in airborne sources, due to well-documented acidification and calcium depletion in soils.

It has been claimed that controlling phosphorus inputs to some estuaries has not been effective in decreasing eutrophication. It seems possible that phosphorus-saturated sediments and catchment soils may greatly delay recovery loading of phosphorus following reductions in point sources, as is now clearly the case for lakes. In most countries, the largest human populations and the longest and most intensive conversions of catchments have occurred in coastal areas. To return the waters in such catchments to a phosphorus-limited state may require decades of reduced phosphorus application. I was unable to find long-term, ecosystem-scale evidence that controlling nitrogen, either alone or in addition to phosphorus, caused oligotrophication of estuaries.
